# Emerging Trends and Technologies Used for the Identification, Detection, and Characterisation of Plant-Parasitic Nematode Infestation in Crops

**DOI:** 10.3390/plants13213041

**Published:** 2024-10-30

**Authors:** Top Bahadur Pun, Roniya Thapa Magar, Richard Koech, Kirsty J. Owen, Dante L. Adorada

**Affiliations:** 1 School of Engineering and Technology, Central Queensland University, Rockhampton, QLD 4701, Australia; 2 DOE Joint Genome Institute, Lawrence Berkeley National Lab, Berkeley, CA 94720, USA; 3 School of Health, Medical and Applied Sciences, Central Queensland University, Bundaberg, QLD 4760, Australia; r.koech@cqu.edu.au; 4 School of Agriculture and Environmental Science, University of Southern Queensland, Toowoomba, QLD 4305, Australia; 5 Centre for Crop Health, University of Southern Queensland, Toowoomba, QLD 4305, Australia

**Keywords:** plant-parasitic nematodes, morphological identification, molecular diagnostics, deep learning, hyperspectral imaging, remote sensing

## Abstract

Accurate identification and estimation of the population densities of microscopic, soil-dwelling plant-parasitic nematodes (PPNs) are essential, as PPNs cause significant economic losses in agricultural production systems worldwide. This study presents a comprehensive review of emerging techniques used for the identification of PPNs, including morphological identification, molecular diagnostics such as polymerase chain reaction (PCR), high-throughput sequencing, meta barcoding, remote sensing, hyperspectral analysis, and image processing. Classical morphological methods require a microscope and nematode taxonomist to identify species, which is laborious and time-consuming. Alternatively, quantitative polymerase chain reaction (qPCR) has emerged as a reliable and efficient approach for PPN identification and quantification; however, the cost associated with the reagents, instrumentation, and careful optimisation of reaction conditions can be prohibitive. High-throughput sequencing and meta-barcoding are used to study the biodiversity of all tropical groups of nematodes, not just PPNs, and are useful for describing changes in soil ecology. Convolutional neural network (CNN) methods are necessary to automate the detection and counting of PPNs from microscopic images, including complex cases like tangled nematodes. Remote sensing and hyperspectral methods offer non-invasive approaches to estimate nematode infestations and facilitate early diagnosis of plant stress caused by nematodes and rapid management of PPNs. This review provides a valuable resource for researchers, practitioners, and policymakers involved in nematology and plant protection. It highlights the importance of fast, efficient, and robust identification protocols and decision-support tools in mitigating the impact of PPNs on global agriculture and food security.

## 1. Introduction

Nematodes are ecologically diverse, abundant metazoan organisms that inhabit soils, sediments, or host plants and animals [1,2]. Although millions of nematode species inhabit the natural world, only around 30,000 species have been explored so far [3]. Among them, 4100 species are identified as PPNs [4]. Many PPNs are destructive pests and are significant threats to crop production. They alter crop physiology, suppress plant immune responses, and allow secondary infection by pathogens [5].

PPNs are broadly classified into two groups based on their parasitism: ectoparasites and endoparasites. Ectoparasitic nematodes feed on the outer plant tissues from the root surface and remain outside the host plant, whereas endoparasitic nematodes enter the host plant and damage the cells and tissue layers [6,7]. The most damaging PPNs are the sedentary endoparasitic nematodes, such as root-knot nematodes (RKNs) (*Meloidogyne* spp.) and cyst nematodes (including *Heterodera* spp. and *Globodera* spp.). Although plant cell walls contribute to mechanical support and rigidity, they can also provide a physical barrier against the pathogen. Nematodes secrete enzymes from pharyngeal gland cells that weaken the cell wall and help to penetrate the root tissue using stylet [8]. Despite the differences in PPNs’ feeding habits, all species penetrate the outer cell wall to access the cell cytoplasm to feed [6]. Consequently, plant root systems are unable to absorb sufficient nutrients and water [9]. The symptoms of nematode infestation can lead to nutritional deficiencies or exacerbation of abiotic stresses, resulting in reduced growth, wilting, and poor yield in intolerant plants [10].

PPNs cause economic losses of more than USD 215 billion worldwide [11,12]. Accurate identification of PPNs is essential in order to devise appropriate strategies to control or minimise their impact on crops. It is equally important to quantify the potential magnitude of the damage by estimating the PPN population densities in soil and/or plant roots. Traditionally, nematologists identify PPNs based on morphological features using a microscope. Additionally, there are several identification techniques based on molecular and biochemical characteristics of the nematodes.

**Figure 1:**
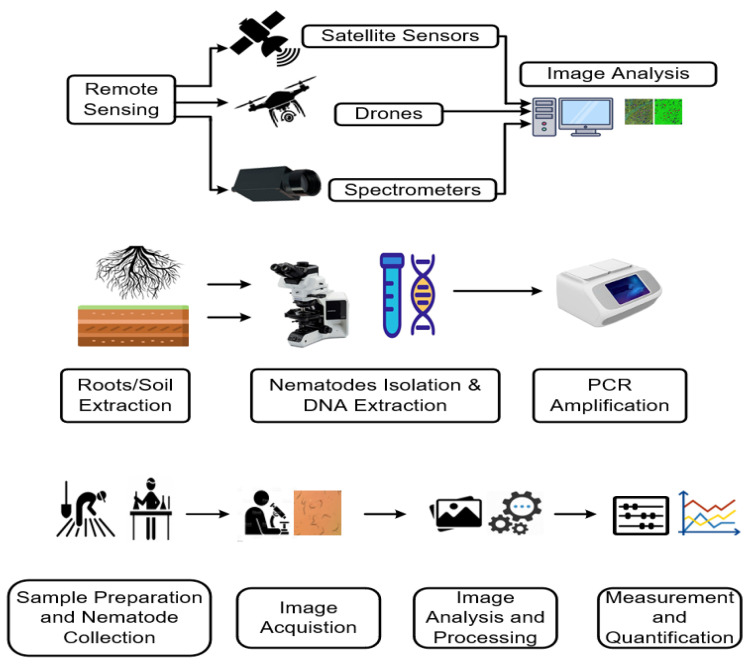
Workflow of remote sensing, polymerase chain reaction (PCR) and image analysis methods to detect and count plant-parasitic nematodes.

To the best of our knowledge, most of the published reviews on this topic have focused on morphological and molecular techniques. Recently, there have been several new studies published that refined previous methods or provided novel techniques for nematode identification. This review investigates current and emerging technologies used for nematode identification and quantification, including morphological, biochemical, and molecular techniques, as well as image analysis, deep learning, hyperspectral, and remote sensing (Figure 1). Furthermore, it provides analytical insights into the significant outcomes achieved by these different methods.

## 2. Nematode Identification Methods

### 2.1. Nematode Identification Using Morphological Methods

**Figure 2:**
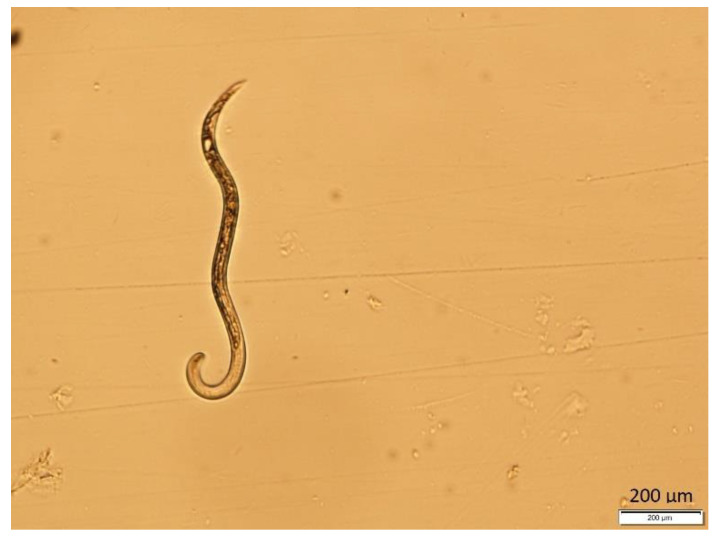
Sample image of a root-knot nematode.

The traditional methods of nematode identification require a microscope and trained nematologists to observe the morphological characteristics of nematodes and determine their genus and species. Nematologists differentiate nematode species in terms of morphological features such as the length and width (Figure 2).

Furthermore, the discriminating body parts include the anterior or posterior region of the body, tail shape and stylet morphology [13]. For example, female RKN species can be identified using the distinguishing features of the head and stylet of second-stage juveniles (J2). The J2 head shape, length between dorsal oesophageal gland orifice (DEGO) and stylet base, and stylet morphology such as the length of the cone, shaft, and knobs are useful for diagnosing morphological characteristics [14].

In a past study, the morphology of cyst nematodes (*Heterodera elachista*, *H. oryzicola*, *H. oryzae*, and *H. sacchari*) was differentiated based on the length and width of the cyst, vulval cone, juvenile stylet length, tail length, and body length [15], which are universal features of the *Heterodera* genus and *Globodera pallida*. Similarly, potato cyst nematodes (*G. pallida* and *G. rostochiensis*) were distinguished by examining the morphological features of second-stage juveniles (J2), including parameters such as the J2 body length, body width, stylet length, and stylet knob structure [16].

Many morphological features are also crucial for understanding the physiological functions and interactions of nematodes with the environment and plant hosts [17]. In addition to morphological features, anatomical elements and the presence or absence of male nematodes are also considered. Morphological identification of nematode features using a microscope is time-consuming and error-prone because of variations in the morphology within species [18]. Moreover, the shortage of nematode taxonomists has led to reliance on molecular-based nematode identification methods. Consequently, molecular methods have become essential for discriminating among nematode species [19].

### 2.2. Identification of Nematodes Based on Biochemical Methods

Biochemical methods involve distinguishing proteins and isozymes using one- or two-dimensional gel electrophoresis analysis or serological analyses. This type of multilocus enzyme electrophoresis, also called isoenzyme typing, is based on isozyme migration patterns related to the molecular weight, electrical charges and variation in the amino acid composition [2,20]. The isozyme phenotypes were first used to differentiate *Meloidogyne* species (*M. arenaria*, *M. hapla*, *M. incognita*, *M. javanica*) [21]. The carboxylesterases/esterases were found to be efficient enzymes for discriminating these species. In addition, the malate dehydrogenase, superoxide dismutase, and glutamate oxaloacetate transaminase are regularly used to identify *Meloidogyne* species. This technique has been used to investigate how nematodes can adapt and evolve in reaction to the changes in their environment [22]. However, this method requires a sufficient amount of isozyme [23], along with various chemicals (creatine phosphokinase, lactate dehydrogenase) and techniques (gel electrophoresis, staining), to obtain results from multiple isozymes [24]. As an alternative to this method, molecular techniques such as DNA sequencing and polymerase chain reaction (PCR) have been reported to be more efficient, as they use a single process for all the DNA markers [24,25]. These molecular technologies are now critical for the diagnosis, treatment and control of multiple PPNs [26].

### 2.3. Identification of Nematodes Using Molecular Methods

Molecular testing has emerged as one of the most widely used methods for the identification and quantification of PPNs. These methods have high sensitivity and can distinguish between morphologically similar organisms, and they are effective in detecting PPNs in asymptomatic infestations or those with a low parasitic burden [27]. However, these techniques require molecular diagnostic tools, reagents and sophisticated facilities [28]. The main benefits of molecular diagnostics are that they can provide fast, accurate, high-throughput potential with readily available sequencing information, which can account for the phenotypic variation and nematode growth stages [29,30].

Molecular diagnostics have often used two primary genomic regions as specific targets for sequence divergence, ribosomal RNA (rRNA) genes and the mitochondrial cytochrome oxidase subunit I (COI) gene [31]. The rRNA genes include the coding genes 18S, 5.8S and 28S with non-coding internal transcribed spacers (ITS1 and ITS2). These genes form a region of the genome that is highly conserved yet divergent enough to effectively distinguish between species among various nematode groups. These genes are present in multiple copies in genomes, which facilitates their amplification using polymerase chain reaction [32]. The COI of the mitochondrial DNA (mtDNA) has been widely used as a standard barcode marker for the identification of metazoans. The mtDNA forms the region of the genome sequence ranging from 12 to 20 kilobases [33]. Variations in mtDNA sequences serve as effective markers to distinguish between nematode species [34]. Similarly, satellite DNA (satDNA) was amplified to identify *M. hapla* using DNA extracted from juveniles and eggs, which can be implemented for a routine-based diagnostic tool [35]. Further, a species-specific probe satDNA sequence was used to detect *Pratylenchus thornei* and evaluate the diagnostic potential as it offers an alternative approach for specific identification that does not require PCR amplification of DNA [36]. In addition, heat shock protein (HSP) was used to isolate and characterise the cDNA and corresponding genes of *M. artielllia*, which could be beneficial for studying multiple sensory behaviours during the development and survival of different stages of the nematode [37]. HSP90 was utilised to explore the relationship between the survival of *Bursaphelenchus xylophilus* and the functionality of the HSP90 gene [38].

Besides the target regions, several marker-based methods are used to identify PPNs, such as restriction fragment length polymorphisms (RFLPs), amplified fragment length polymorphism (AFLP), random amplification of polymorphic DNA (RAPD), and sequence-characterized amplified region (SCAR). The RFLP method analyses include amplifying a target region and then digesting it with restriction enzymes. The DNA fragments resulting from the restriction analysis are subsequently separated by size through gel electrophoresis [39].

AFLP involves digesting DNA with restriction enzymes, selectively amplifying a subset of DNA fragments and separating them by electrophoresis on a polyacrylamide gel [40]. This technique is particularly useful for identifying species with incomplete genome sequences [41]. AFLP is reported to be a highly reproducible and robust tool for species identification and phylogenetic analysis [41]. The RAPD method amplifies random DNA segments using arbitrary primers [42]. It is a simple and cost-effective method for studying biodiversity; however, it requires a consistent amplification protocol to ensure sample reproducibility [43]. Furthermore, SCAR is a reliable method for generating DNA fragments amplified by PCR [44]. SCAR-based markers are used for identification with both traditional and real-time PCR methods [18,21,33]. The SCAR marker was developed to identify the *M. ethiopica* nematode species in field samples [45]. These methods require a basic molecular biology laboratory with major facilities such as a PCR machine, refrigerator and freezer, DNA sequencer, high-quality reagents, gel electrophoresis, microcentrifuge, computational tools, and a sterile work environment. The advantages and disadvantages of these markers are described in Table 1.

### 2.4. Next-Generation Sequencing/Deep Sequencing

Next-generation sequencing (NGS) is an innovative technology utilised for sequencing DNA and RNA, as well as detecting variants and mutations. NGS has the capability to quickly sequence hundreds or thousands of genes or even entire genomes [65]. It can simultaneously analyse numerous DNA targets, ranging from hundreds to potentially millions. For instance, NGS has been used to investigate the effects of nematode communities and to analyse the sustainability of banana and coffee soil ecology [66]. Next-generation sequencing (deep sequencing) has been used to identify the microRNA genes of RKN [67]. Similarly, the microRNA of soybean (*Glycine max*) is utilised in the identification of soybean cyst nematodes (SCN, *H. glycine*) through deep sequencing [68]. Some of these miRNAs potentially target stress-responsive genes. In addition, a wide range of small RNAs respond to SCN infection in both resistant and susceptible soybean roots. NGS has great potential for exploring parasitism in nematodes [67,69]. However, the limited availability of suitable primers and extraction kits poses a challenge for their use in DNA analysis [69]. Nevertheless, a recent study developed different primers suitable for identifying various species of nematodes using NGS-based metabarcoding [70]. NGS has been used to diagnose *M. incognita* and *M. javanica* infestation in tomato [71].

### 2.5. DNA Metabarcoding

Genetic markers in barcoding and metabarcoding analyses enhance the taxonomic assignments at the genus level [66] and offer significantly improved precision of identification at the species or genus level, in combination with molecular and morphological analyses [72]. Metabarcoding allows improved precision of identification of soil nematode communities [73,74]. For example, the DNA barcoding method was used to identify species of *Heterodera* [75], where combined markers optimised the results. Moreover, another study focused on the investigation of new and existing primer sets for the metabarcoding of plant-parasitic nematodes and free-living nematodes [76]. Although metabarcoding is widely used for classifying nematode species, it often fails to detect all the taxa present in a sample because PCR primers do not bind effectively to their target [77]. A mitochondrial metagenomics (mtMG) approach was used to investigate the diversity of nematode species that were also evaluated morphologically to identify nematode species in terms of the feeding habits, phylogenetic relationships, and life stages. However, this study also found a limited reference database. Another study investigated the mitochondrial and ribosomal reference sequences to determine the species-level clustering threshold, reporting it to be the most reliable method for rapidly assessing the alpha diversity in environmental samples [78]. Further, the meiofaunal diversity of PPNs was explored in Atlantic soil using eukaryotic metabarcoding analysis [79]. This study revealed that the combination of high-throughput sequencing and morphological analysis can resolve taxonomic classification discrepancies and serve as a robust tool for investigating the biological diversity and conservation management of soil species.

### 2.6. Nematode Identification Using Polymerase Chain Reaction (PCR) Methods

Polymerase chain reaction (PCR) is one of the most powerful tools used for the analysis of DNA sequencing in various fields of molecular biology research [80] and it has been widely used in the identification of PPNs [81], phylogenetic studies, plant resistance, and gene studies [31]. It uses several primers to identify nematodes causing the infection based on targeted regions in the genome, such as ribosomal deoxyribonucleic acid (rDNA), SCAR, ITS, intergenic spacer region (IGS), and satellite DNA (satDNA) [82]. The relevant studies on the identification and quantification of PPNs are shown in Table 2.

PCR-based methods were employed to detect *M. arabicida* and *M. izalcoensis* in soil and root samples of coffee using SCAR markers [25]. These markers were an effective alternative species-specific molecular marker. Further, a species-specific SCAR marker was developed to identify *M. ethiopica* in kiwi fruit [45], where the specificity of the primer pairs was validated by analysing other RKN species. Isolates of *M. ethiopica* were considered complementary to esterase phenotyping for identifying nematodes, offering simple and fast detection of *M. ethiopica*. Similarly, sets of SCAR primers were developed to amplify the DNA of *M. chitwoodi*, *M. fallax* or *M. hapla* species [52]. These SCAR markers were readily detected in DNA samples extracted from juveniles, egg masses, or females of the specific nematode species, whether the nematodes were found individually, mixed with other species, or within infested plant material. However, these SCAR primers were unsuitable for use in a multiplex PCR assay, as the SCAR markers for one or two species were either not amplified or were barely detectable. To address this issue, a new optimized multiplex PCR was used, but it could not visualize DNA extracted from a single juvenile [52]. The study reported that using nested primers significantly enhanced the sensitivity in the subsequent PCR, suggesting potential applications for PPN diagnosis using DNA extracted from single juvenile, soil samples, or infected plant material.

Real-time PCR quantification of PPNs using specific primers for DNA synthesis has been developed for RKNs (*M. incognita*), root-lesion nematodes (*P. penetrans*), potato cyst nematodes (*G. rostochiensis*), and soybean cyst nematodes (*H. glycines*) [83]. The nematodes were extracted from roots and soil by the Baermann funnel extraction method and real-time PCR was used to determine the density of the nematodes (egg, juvenile or adult stages) in radish (*Raphanus sativus*), sweet potato (*Ipomoea batatas*), and lotus (*Nelumbo nucifera*). Although a strong correlation was found between the number of *M. incognita* using the Baermann method and the PCR method, there was a significant increase in the density of *P. penetrans*, which was likely caused by the presence of the DNA of dead nematodes.

The root-lesion nematode, *P. thornei*, was detected and quantified in wheat using the asymmetrical cyanine dye (SYBR green-I)-based PCR method [84]. In this study, the Whitehead tray method was used to extract nematodes from soil samples and a PowerSoil DNA Isolation kit was used for DNA extraction directly from the soil. A designed primer set was used in the ITS region of the rDNA for real-time PCR. The study found a strong correlation between the number of *P. thornei* counted using a microscope and the population determined by the PCR method. Extraction of DNA directly from the soil eliminated the manual extraction process and need for counts using a microscope.

Discrimination of mixed populations of RKNs (*M. incognita*, *M. javanica*, and *M. arenaria*) by qPCR was determined by first extracting nematodes from field soil using the sugar centrifugation method [85]. There was a high correlation between the qPCR assay and counts using a microscope. However, in some cases, high cycle threshold (Ct) values were found to limit the detection results. Real-time PCR was used to detect and quantify root-lesion nematodes (*P. vulnus*) and ring nematodes (*Mesocriconema xenoplax*) in a walnut (*Juglans regia*) and almond (*Prunus dulcis*) orchard [86]. In the samples from walnut, the qPCR method accurately detected nematodes and the study found a strong correlation (coefficient of determination (R^2^) = 0.88 for *P. vulnus* and R^2^ = 0.65 for *M. xenoplax*) with the counts using a microscope. In samples from the almond orchard, the qPCR predictions were highly correlated with the counts using a microscope (R^2^ = 0.87 for *P. vulnus* and R^2^ = 0.90 for *M. xenoplax*). The study also revealed that the efficacy of the molecular assay was reduced with relatively low organic matter (0.6–0.9% in almond orchard soil and 1.8% in walnut orchard soil) and low clay composition because of the binding with the extracted DNA. Another challenge was the inconsistency in the soil quantity when estimating nematode populations using molecular diagnostics, as it was unclear what amount of soil accurately represents these populations. A recently developed PCR technique, droplet digital PCR (ddPCR), has shown considerable sensitivity in detecting and quantifying the stubby root nematode, *Paratrichodorus allius*, in soil [90]. The method (ddPCR) eliminates the need for manual nematode extraction, microscopic identification and quantification of the Ct values because it provides direct copy numbers. ddPCR is resistant to PCR inhibitors and is effective for the absolute quantification of low-abundance targets; however, it does have a limited dynamic range.

### 2.7. DNA Microarray

DNA microarray, also known as DNA chips, is a commonly used method for analysing global gene expression and assessing microbial diversity [91,92]. It is based on nucleic acid hybridisation, where the target sequences are typically labelled with fluorescence and hybridised to spots of complementary oligonucleotide probes fixed to a solid surface [93]. A DNA oligonucleotide microarray was used to identify *M. chitwoodi* in pure and mixed samples [94]. The main advantage of the DNA microarray method is that it can be used for large-scale investigations without the need for the isolation of nematodes [95]. Nevertheless, the DNA microarray is expensive, time-consuming, and requires expertise in molecular technology [2,95]. The signal measured on a microarray might not be consistent over a range of concentrations in a solution, making it cumbersome to detect every gene among multiple related genes [96]. In contrast, NGS offers many benefits over the DNA microarray method. Sequencing is an unbiased method for determining the nucleic acids present in a solution, as it does not rely on prior knowledge of their presence [96]. It can also identify closely related gene sequences that might be undetected due to cross-hybridisation on DNA microarrays [96]. As an alternative to these molecular methods, nematodes can be detected and enumerated by analysing images taken using a microscope. Several methods are used to study the morphology of nematodes using image analysis and computer vision [97,98].

### 2.8. Matrix-Assisted Laser Desorption/Ionisation Time-of-Flight Mass Spectrometry (MALDI-TOFMS)

Matrix-assisted laser desorption/ionisation (MALDI) is one of the most popular methods for identifying biomolecules [99]. The specific method, matrix-assisted laser desorption/ionization time-of-flight mass spectrometry (MALDI-TOFMS), was first applied to detect a single root-knot nematode (*M. incognita*) [100]. The method was found to be rapid and sensitive in detecting a single nematode and capable of differentiating between the infective and non-infective stages of nematodes. Additionally, PPNs have been identified based on the analysis of the nematode protein profiles using MALDI-TOFMS. The findings indicated that the protein profiles were effective in the identification of PPNs [101]. This method is simpler and faster for detecting PPNs in mixtures compared to traditional grinding methods.

### 2.9. Isothermal Amplification Technologies

Isothermal amplification is a technique used to amplify specific DNA or RNA sequences that do not require thermal cycling. It is easy to operate and reduces the risk of sample contamination [102]. Loop-mediated isothermal amplification (LAMP) has become a reliable and fast method to detect PPNs [103], such as *Ditylenchus destructor* [104], *Meloidogyne* spp. [105], *M. partityla* [106], and *M. enterolobii* [107]. LAMP has been regarded as an alternative method, which is 10–100 times faster than conventional PCR. However, LAMP is sensitive to cross-contamination and requires tedious steps to check for the presence of reaction inhibitors [108]. In addition to LAMP, the recombinase polymerase amplification (RPA) method has been used to identify *P. allius*, where it required low temperatures and a short duration to amplify DNA [109]. Although RPA methods detect nematodes accurately with high specificity, the method is dependent on the designed primers. Further, the RPA assay is not used to detect DNA extracted directly from soil. However, RPA with lateral flow dipstick was used to detect *H. schachtii* and its sensitivity was higher than PCR and qPCR, providing results within 1 h [110].

### 2.10. Conventional Image Processing Method

Traditional image processing methods are used for the identification and assessment of nematode biomass and growth. Image processing and computer vision approaches are used to analyse microscopic images captured using various types of sensors. Nematode experts initially identify distinct features such as the body shape and size and other morphological characteristics to distinguish different genera of PPNs. These discriminant features are integrated into the image processing algorithms, the automated identification process, and the computation of the nematode population. Several studies have explored image analysis methods, as shown in Table 3.

A semi-automated image analysis method used the length and width of nematodes to estimate the biomass of meiofaunal nematodes using the curve perimeter and curve area [114]. The image analysis method computed similar lengths as measured by humans; however, significant differences were found in the nematode widths measured by humans and computers. Automatic nematode width measurement was found to be two times faster than manual measurement.

Similarly, the length and width of the RKN were assessed for the detection and quantification of RKN populations using image processing and computer vision [97]. A new method was proposed to detect and count RKN juveniles using threshold segmentation [97], employing non-linear morphological operations to remove soil particles. The algorithm implemented the length-to-width ratio to distinguish between nematodes and other substrate particles and found the highest correlation between the manual and automated computation of the nematode length compared to a biomass study [114]. Further, it specified the optimal length-to-width ratio to detect nematodes. However, this method was unable to detect and count overlapped nematodes, which posed a significant challenge when dealing with increasing nematode population density within the specimens. Moreover, few studies have investigated nematode infestation in terms of the nematode eggs [115,116,117], root gall index [118], and number of adult females [112,113]. A new feature was used to detect and count RKN eggs in images from microscopes [115] using the difference between the middle width of the nematode egg and the average width of the egg to assist in automating the detection task. However, the applicability of these features may vary according to the nematode species. These features may vary due to several factors, such as differences in the morphological characteristics, genetic variation, and environmental adaptation.

In addition, some research has focused on the detection and counting of female PPN populations using a scanner rather than a microscope. For the development of a high-throughput method to count female *H. glycines*, cysts were extracted from soybean (*Glycine max*) roots. There was a high correlation between the automated and manual counting and the high-throughput method was two times faster than the manual counting method; however, it required additional steps to process the roots [112]. Further, a similar study on cereal cyst nematodes (*H. avenae*) based on image analysis techniques identified *H. avenae* females in soil and extracted from roots using a scanner [113]. The naïve Bayes classification had a 96.1% accuracy rate and claimed to be a simple method that could be applied to different complex backgrounds.

### 2.11. Nematode Detection Using Deep Learning Methods

Deep learning is a subfield of an emerging machine learning method whose architecture is inspired by human brains when analysing microscopic images and segmenting cells and tissues [119,120]. There are different types of deep learning models, such as convolution neural network (CNN) [121], recurrent neural network (RNN) [122], deep feedforward neural network (DFNN) [120] and autoencoder [123]. CNN is the most popular deep learning architecture used for biomedical image analysis [124]. All these deep learning models have a hierarchical structure consisting of three layers of convolution, pooling and fully connected layers [125,126]. Deep learning can be trained through forward and backward stages. The goal of these networks is to adjust the weight and bias based on gradients to compute the loss function. During this phase, the network learns features required for the detection and classification of objects. After sufficient training, the network learning process can be concluded. The performance of the model is evaluated on test data to verify its effectiveness on unseen data. Deep learning models are employed to detect microscopic images due to their speed and accuracy [120]. They are also used to detect and count bacterial colonies [127], protozoan parasites [128] and cells in microscopic images [129]. Recently, deep learning models have been used to detect and enumerate nematodes in microscopic images (Table 4).

A convolutional neural network (CNN)-based deep learning model was developed to identify *Globodera* species [131]. The datasets consisted of 360 images of *Globodera* species, which were later augmented to 14,490 using colour channel extraction. The study also used a custom computer vision algorithm (CCVA) to identify the morphological features of the basal knobs width (BKW) and basal knob to head length (BKTH). The EB-Net architecture was used in the CNN model to extract morphological features. The BKTH feature was predicted appropriately, whereas the BKW feature was poorly detected by the computer vision method and EB-Net model. The EB-Net model predicted metrics with 0.83 accuracy on the training set, whereas the CCVA method predicted metrics with 0.88 accuracy. The CCVA method achieved 0.85 accuracy on the test set; in contrast, the EB-Net model achieved 0.71 accuracy.

A web-based NemaRec application was developed to identify nematodes in microscopic images [132] using image data from the I-Nema dataset [137]. Further, Gaussian blurred and random flip methods were used to create an augmented dataset. The ResNet-101 model was used to identify the genera of nematodes and showed that the ResNet-101 model identified 60% of genera accurately in the specimen-based dataset and 94% to 97% of genera in the augmented dataset.

Another study was undertaken to identify *Helicotylenchus dihystera*, *H. glycines*, *Meloidogyne* sp., *P. brachyourus*, and *Rotylenchulus reniformis* from soybean [133]. The dataset used consisted of 3063 microscopic images of the nematodes taken with a 16-megapixel Panasonic camera attached to the light microscope with 5x objective lenses. The images were preprocessed to remove any geometric distortion, aliasing, noise, shading, and photometric nonlinearity. To extract the features and classify the nematodes, a CNN model was customised using DenseNet121 with multiple inception blocks. The image data were augmented using the random transformation of mirroring, rotation, and flip methods to avoid overfitting and improve the generalisation error. The model was optimised with stochastic gradient descent. NemaNet revealed the highest accuracy compared to InceptionV3, Xception, InceptionResNetV2, and DenseNet169 while training from scratch and using transfer learning.

The root galls of the RKN in cucumber (*Cucumis sativus*) were detected using the YOLOv5-CMS model [134]. A Canon EOS 60D camera was used to acquire 686 images of the root galls and the final images were resized to 640 × 640. These image data were augmented with random rotation, random brightness, and random contrast. The YOLOv5-CMS model was enhanced with a dual attention module to extract important features of the root galls image. The K-means ++ algorithm was implemented in place of the K-means algorithm to cluster the bounding box and obtain the anchor box size. The original YOLOv5 model used the Complete Intersection over Union (CIoU) loss function, which was replaced by the SCYLLA-Intersection over Union (SIoU). The experiment showed that YOLOv5-CMS achieved the highest accuracy, with precision = 94.3%, recall = 88.5%, F1-score = 91%, and mAP = 94.8%, compared to the faster recurrent–convolutional neural network (R-CNN), original YOLOv5, YOLOv3, and YOLOv4. The YOLO model performed well in the real-time object detection problem; however, the object localization accuracy has been a challenging issue for the YOLO model [138]. To investigate these issues, YOLO models have also been used to detect and quantify nematodes [135]. This study revealed that the YOLOv5 model attained the highest accuracy in the classification and counting of nematodes compared to other models. This study also investigated the performance of YOLO models in detecting nematodes that overlapped each other in the microscopic images and showed that the YOLOv7 model performed better than other models in that regard. This study also highlighted the significant application of mosaic augmentation in analysing microscopic images.

The deep learning models are further used for detecting nematodes eggs. In addition, a deep learning-based decision-support tool was built to estimate the nematode eggs and juvenile populations in microscopic images and recommend optimal nematode management strategies in terms of a damage threshold [136]. This tool can estimate nematode juveniles and eggs in microscopic images and allow the recording of population information in a database to track population growth for future studies and nematode management recommendations.

Furthermore, soybean cyst nematode eggs were estimated using deep learning [139]. The method used a convolutional selective autoencoder-based deep learning model to detect and count soybean cyst nematode eggs in microscopic images. This robust method reported counting one frame per minute in highly cluttered images and 242 frames per minute in less-cluttered frames. In another study, robotic instruments were developed to automate the process of SCN egg extraction [140], contributing to decisions regarding integrated pest management. Thus, different types of state-of-the-art technologies have been employed to automate nematode detection and enumeration processes.

### 2.12. Nematode Infestation Detection Using Hyperspectral Imaging

Hyperspectral imaging uses spectroscopy and radiometric methods to observe the biological sample response at the molecular level when exposed to light [141]. This method can detect chemical constituents, defects, and contamination of food or agricultural samples. The spectral signature captured at different growth stages of crops can be used to analyse crop health conditions [142]. Few studies present the detection of nematode infestation via hyperspectral interventions. Hyperspectral data were used to detect early infestation of RKN in cotton [143]. This study used a machine learning classifier, linear discriminant analysis (LDA), principal component analysis (PCA), and stepwise LDA to classify RKN-infested and non-infested plants. The machine learning classifier, PCA and SLDA found a spectra range of 350–1000 nm appropriate for detecting RKN infestation with overall 95% classification accuracy.

Hyperspectral data were used to differentiate between RKN infestation (biotic stress) and water deficiency (abiotic stress) in tomatoes [144]. This study used HySpex VNIR and SWIR spectrometers (HySpex, Oslo, Norway) with two halogen light sources to capture hyperspectral data. The spectra ranging from 400 to 2500 nm were analysed through partial least square discriminant analysis (PLS-DA) and a PLS support vector machine (PLS-SVM). The PLS-SVM identified RKN infestation with 90–100% accuracy. It also discriminated between water deficiency and well-watered plants with 100% accuracy. The short-wave infrared spectra were linked to O–H and C–H stretches and were most important for the detection of nematode infestation and the severity of that infestation.

### 2.13. Nematode Infestation Detection Using Remote Sensing

Remote sensing has recently been used in agriculture to investigate the temporal and spatial variation of crop morphology and physiological conditions [145]. Remote sensing is a sensor technology that utilises a range of electromagnetic radiation to acquire physical data about an object without contact [146]. Hyperspectral remote sensing involves capturing images of the object at different bandwidths of the wave spectrum and facilitating the identification of properties that are invisible to a particular bandwidth [147]. Recently, remote sensing with hyperspectral imaging has been used to detect nematode infestation. Narrow-band sensors can capture a rich set of data that can be used to analyse the physical and chemical characteristics and identify the proteins and metabolites triggered by the immune system of plants [148]. Examples of studies undertaken to detect nematodes using remote sensing are summarised in Table 5.

Nematode infection in roots causes the spectral variation of leaves, as demonstrated by a study that used remote sensing to detect RKN infection in coffee crops [149]. A RapidEye sensor was used to acquire hyperspectral or radiometric data. In addition, the biomass content, leaf area index (LAI), and chlorophyll content collected from a spoil and plant development analyser (SPAD) were used to discriminate healthy, moderately infected, and severely infected plants. This study found that the LAI, biomass content, and SPAD could not differentiate between healthy and nematode-infected coffee plants. In contrast, red, near-infrared, and red-edge spectra are advantageous in differentiating healthy and infected coffee plants. The Normalised Difference Vegetation Index (NDVI) attained the highest classification accuracy of 78%, with a kappa coefficient of 0.71, in differentiating early and severely infected coffee plants.

The NDVI has also been used to discriminate between wheat cultivar tolerance and resistance responses to *P. thornei* in field experiments [160,161]. The NDVI, which is the difference between the reflectance of near-infrared and red wavelengths, measures plant greenness. The tolerance of wheat cultivars at 1000-degree days after sowing in a field with damaging population densities of *P. thornei* was highly correlated with the grain yield (R^2^ = 0.92). The degree days are calculated by averaging the minimum and maximum temperature to compute the cumulative thermal time in degree days above a baseline temperature (0 °C) [160]. Similarly, wheat cultivar resistance to *P. thornei* in field experiments was also highly correlated with glasshouse-derived ratings of resistance [161]. Remote sensing with the NDVI has the potential to allow plant breeders to rank and select germplasm for tolerance and resistance in a high-throughput field-based system. In another study, remote sensing was employed to assess nematode (*H. schachtii*) and fungal (*Rhizoctonia solani)* infection in sugar beet fields [150]. Hyperspectral data were taken from an Airborne Imaging Spectroradiometer for applications and near-range spectral data were collected using an ASD FieldSpec Pro Spectrometer (Malvern Panalytical, Malvern, UK) from one metre above the canopy. A Hyperspectral Mapper imaging sensor was utilised to acquire 126 spectral bands between 450 and 2500 nm. The spectral angle mapper was used to classify the hyperspectral images and generate the digital map. The important spectral vegetation indices, the NDVI, water index (WI), chlorophyll content (CC), simple ratio pigment index (SRPI), and structural independent pigment index (SIPI), were correlated with the leaf and beet weight measurement and population density. The results showed that the spectral vegetation indices were highly correlated with beet cyst nematodes in wet and mild temperatures. The spectral angle mapper (SAM) method attained a classification accuracy of 72%, with a kappa coefficient of 0.65. Remote sensing in hot and dry seasons requires careful consideration when detecting nematodes. The inverse distance weighting interpolation method employed in digital maps is a powerful tool to differentiate the spatial distribution. A disparity in the spectral and spatial resolution can result from inconsistency in nematode infection detection.

The infestation of pine trees by *B. xylophilus* causing pine wilt disease (PWD) has also been identified using remote sensing and geographical positioning system (GPS) data [151]. Field drone images of pine trees were acquired using a DJI Phantom 4V 1.0 (DJI, Shenzhen, China) at two locations in Republic of Korea (Anbi and Wonchang) and GPS data were collected from a Garmin Oregon 750T. The images were classified into normal pine trees, PWD-infected trees, bare land, roads, shadows, and grass fields. This study employed an artificial neural network (ANN) and support vector machine (SVM) to classify the images. The classification accuracy was computed with a stratified method based on pixels. Random pixels were selected from each class in the drone image and used as reference points, then compared with SVM and ANN classifiers. The SVM classifier achieved the highest accuracy of 94.13% on the Anbi data and 86.59% on the Wonchang data, whereas the ANN classification performance was slightly lower with 87.43% on the Anbi data and 79.33% on the Wonchang data.

### 2.14. Detection of Plant-Parasitic Nematodes in the Field

In order to effectively implement field testing, it is important to assess the feasibility of field operations that encompass the entire detection process, including sample handling, the amplification process, and result visualisation. PPNs can be identified in the field using in-field compatible technology, such as a solid-phase-based method for DNA preparation developed by Flinders Technology Associates (FTA) that can be carried out at room temperature [162]. This method simplifies the field sampling and genomic analysis of PPNs. Similarly, the FTA method was combined with LAMP to detect *M. hapla* in the field [163]. This combined approach used a non-toxic reagent and reduced the detection time by one hour when identifying nematodes in the field. This method also has high sensitivity and resistance inhibitors such as humic acid and proteins. In addition, DNA extraction kits are also available to extract DNA directly from soil. For instance, PowerSoil Pro Kits were used to extract the DNA of *P. allius* and *M. incognita* for quantification in field tests [85,90]. However, the accuracy of in-field tests using kits may reduce the detection efficiency due to the non-uniform distribution of nematodes in soil [164].

## 3. Discussion

PPNs are diverse species, with each showing a unique response to environmental factors. Different methods are utilised to detect and enumerate PPNs, with the most predominant one being manual counting with the aid of a manual microscope. Morphological identification and image analysis require accurate nematode extraction, particularly for nematode population enumeration. The optimal extraction method would achieve 100% efficiency in extracting all the stages of nematodes species regardless of the temperature and soil type, while taking a shorter time and lower cost in terms of labour and equipment. The nematode extraction time may vary depending on the sieve size, specific density of the nematode and/or the extraction process (sedimentation, flotation/centrifugation, elutriation). Nematologists discern the morphological features of nematodes using a microscope; however, identifying morphological features for long periods can cause eye stress and fatigue. Besides traditional morphological identification methods, nematodes are detected using microscopy image analysis methods. Nematode assessment using image analysis methods achieves a good correlation between manual and automated measurements of length [114], but there can be a non-significant relationship between manual and automated measurements of width. This difference is due to inaccuracy in selecting the widest point on the nematode body. The automated counting method facilitates the estimation of the prediction error, which is not accounted for in the manual counting process [165].

In addition, the automated counting method saves time compared to manual counting. The automated fluorescence-based counting method was reported to be 50% faster than the manual counting method [112]. Nevertheless, the high-throughput counting method was only able to count single species [165] and could not differentiate between dead and live nematodes. The skeleton analysis method could discriminate between live and dead nematodes; however, false detection was increased when live nematodes had a structure similar to dead nematodes [98]. The detection of overlapped nematodes required additional pixel computation using skeleton analysis. The algorithm uses the junction pixel and analyses the angles between two branches and compares them with the threshold angle to separate overlapped nematodes. In conventional image processing and computer vision methods, images are acquired in different sizes, such as 2048 × 1536 pixels [98], 320 × 240 pixels [111], 1600 × 1200 pixels [97], and 2560 × 1920 [114]. While there is no specified pixel resolution for microscopic images, it is typically determined by the features of the microscope camera. Some microscope cameras, such as the Olympus DP73 and DP75, offer the flexibility to adjust the pixel resolution, providing greater control over acquiring microscopic images.

Recently, state-of-the-art machine learning/deep learning models have been employed to identify nematodes using microscopic image analysis. Deep learning methods employ different convolution neural networks (CNNs) with a variety of input image sizes. Most of the studies used an image size of 224 × 224 [130,132,133,166]. However, one study implemented a larger input size (640 × 640) for deep learning because this study used root-knot nematode galls [134]. Also, Fudickar et al. [167] employed an 820 × 821 input image size acquired from the Raspberry Pi camera. Further, deep learning models often require expensive and high computational resources because of the large number of parameters and high-dimensional input data, limiting their practical application. Nevertheless, various techniques have been used to reduce the computational complexity. For example, downsampling is used to reduce the spatial resolution of the feature map [168]. It can decrease the training time to remove redundant or irrelevant information; however, it can lead to the loss of information for small and imbalanced datasets. For instance, the EfficientNet model used downsampling for the original image to minimise the training time [169]. EfficientNetV2B0- and EfficientNetV2M-based deep learning models achieved the best performance using the RMSProp optimiser with brightness augmentation [130]. Nevertheless, the models could only identify nematodes with perfect shapes and undamaged conditions. Similarly, computer vision-based methods and convolutional neural networks accurately identified *G. pallida* and *G. rostochiensis* [131]. The advantage of the CNN is its rapid construction and adaptability to diverse problems, whereas the computer vision method takes time to build and provides robust performance for species-specific classification. But these approaches require a large volume of sample data to create landmarks. Another study also highlighted the importance of a sufficient amount of training datasets to achieve the best performance from the machine learning model [132]. Training a machine learning model with a transfer learning approach is better than training from scratch [133]. Further, the attention mechanism can be used to improve the capability to extract key features [134]. However, challenges might occur due to negligence in the channel, spatial information, and avoiding local information. These problems can be mitigated using resilient and delicate convolution block attention module–coordinate attention (CBAM-CA) to capture special features of the nematode.

The challenge associated with the detection of overlapped nematodes is the unstable shape and changeable structure. Deep learning models can detect the overlapping of multiple nematodes without causing extra computational burden [98,135]. Nevertheless, deep learning models cannot detect nematodes that fall inside one bounding box [135]. A bounding box is a rectangular box that is used to localise objects in an image. This issue can be solved using different approaches to detect overlapped nematodes, such as soft non-maximum suppression [170] and multiclass methods [171].

In analysing microscopic images, image acquisition plays a crucial role in the identification of nematodes. The acquisition of images relies on the specimens, microscope and sensor features and objective lens. Further, it also depends on the skills and expertise of the microscope operator to utilise the optimal settings of the microscope. Some studies investigated appropriate settings of microscopes and cameras to acquire nematode images [97] and found that it is always preferable to use the optimal settings of the microscope and sensors, such as the light source, magnification, pixel resolution, and contrast, to acquire high-quality microscopic images. This is because most nematodes are transparent and colourless, which may cause challenges during the segmentation of microscopic images or detection of nematodes.

As an alternative to these approaches, molecular diagnostic methods, particularly PCR, have been used to identify and estimate nematode populations in soil samples. Real-time PCR is recognised as an excellent method for the identification and quantification of PPNs [172]. However, the presence of dead nematodes in soil samples hinders the accurate quantification of nematodes using this method [83]. On the other hand, real-time PCR estimation showed a high correlation with counts using a microscope of root-lesion nematodes [84] and *Meloidogyne* spp. [85]. Nevertheless, the presence of inhibitors in soil samples can lead to underestimation in molecular assays [85], and non-specific primer sets can cause secondary amplification, resulting in incorrect estimates of the target DNA [84]. Many studies have reported inconsistencies between real-time PCR and the counts from microscopes [173,174]. Such discrepancies may be due to the uneven distribution of nematodes. The sensitivity of molecular assays can be influenced by soil inhibitors [84], such as clay and humic substances and the historical presence of nematodes [163]. The efficacy of molecular assays can also be reduced in soils with lower levels of organics and clay. While PCR-based techniques and morphological methods can be impractical for large sample volumes, advanced techniques such as qPCR, microfluidic PCR, and next-generation sequencing could be the better alternatives for handling numerous samples [2]. Nevertheless, no particular molecular technique provides comprehensive taxonomic information; therefore, the choice of method depends on the sample characteristics, specific research questions, and available resources [175].

In addition, non-invasive hyperspectral imaging has been reported to be labour- and time-efficient compared to other methods. However, it generates high-dimensional redundant data that require efficient algorithms for data modelling, processing and displaying data and images [176,177]. The high-dimensional data can complicate real-time data acquisition and processing [178]. Further, image acquisition requires a greater time because it does not directly measure the chemical composition or texture of an object [179]. Instead, it acquires a wider range of spectral data from an object and uses computation models to interpret the data, and this process requires calibration. Accurate calibration with a white reference is necessary to account for changes in illumination [177]. The white reference is established under the same conditions as the raw image, using a white surface board that facilitates a consistent reflectance of 99%.

Recently, remote sensing methods have also been used to detect PPN infestation. For instance, spectral bands (near-infrared bands, red, red edge) and the NDVI were employed to classify the spatial distribution of healthy, severely infected, and moderately infected coffee plants. However, the classification results were not reliable because of confusion between bare soil and infection in coffee plants [149]. The study showed an inverse relationship between biophysical characteristics, biomass, and RKN infection [149], but this method could not discriminate efficiently between healthy coffee plants and those with moderate levels of infection. The near-infrared spectral regions (720–1000 nm) are more accurate in discriminating between healthy and infected plants; nevertheless, the spectral response gradually deteriorates in both severely and moderately infected coffee plants. In contrast, in field experiments, the NDVI was highly predictive of wheat tolerance and resistance responses to *P. thornei* [160,161].

The spectral vegetation index (SVI) showed a good association between the symptoms caused by the beet cyst nematode and *R. solani* [150]. Nevertheless, inconsistency in the spectral resolution used in the AISA aerial sensor and aerial HyMap sensor might be a possible cause of the poor correlation between nematodes and SVIs. This resulted in differences in the spatial resolution of sensors. Further, the use of the SVI can omit crucial information from spectral segments because dimensionality reduction was used for easy and faster analysis of spectral information. To avoid these issues, the SAM classification method is suitable and provides a multi-temporal assessment of crop canopies. The SAM method was found to be better in generating detailed maps of diseases in crop fields using a hyperspectral sensor compared to the SVI method. In addition, a differential illumination condition can occur due to the sunlight intensity or topography of the field, which induces darker pixels. In SAM classification methods, darker pixels are rendered along with the same vectors [150]. Thus, it can reduce the sampling point for gathering ground truth data, resulting in a more efficient approach.

An artificial neural network (ANN) and SVM successfully discriminated pine wilt-infected trees from other land covers in drone images; however, roads and buildings were detected in some areas, and bare land and grassland were detected as pine wilt-infected trees [151]. Similarly, the fusion model (YOLOv4 and GoogleNet) was able to classify dead nematode-infected pine trees in the aerial image with high accuracy [153], but the model performance was constrained by the inadequate data volume and shorter pine trees that are hidden in the shadows of larger trees. Furthermore, the remote sensing method required high computing resources for satellite imagery and aerial photographs.

Remotes sensing uses various spectral bands to identify materials and is sensitive to specific conditions. The green and NIR spectral bands are most appropriate for distinguishing symptomatic and asymptomatic plants [152]. Remote sensing can be used to monitor plant growth and the nutrient concentration due to nematode infection, stunting, and above-ground symptoms. However, remote sensing using a UAV is limited to single-time data collection from UAV flights. A robust dataset with greater variability is important for training machine learning models.

In addition, remote sensing is limited by the inadequate resolution (temporal and spatial), non-homogenous illumination problems, and cloudy weather conditions [180]. Some of these can be avoided by using unmanned aerial vehicles, but finding the optimal flight parameters (speed, altitude, camera orientation, flight path) is the main challenge associated with UAV-based remote sensing. UAV sensors are also susceptible to cloudy weather conditions and light incidence [158]. Thus, light sources play a significant role in whether data are collected from remote or microscopic sensors.

All the methods used for identifying nematodes have advantages in some aspects and disadvantages in others. For example, qPCR has proven to be more practical than traditional methods for identifying and quantifying plant-parasitic nematodes; however, the presence of decomposed nematodes in samples can lead to inaccuracies in the quantification. Additionally, factors like clay and humic substances may result in an underestimation of nematode numbers. While deep learning methods can detect and estimate plant-parasitic nematodes more accurately and efficiently than humans, they require a large amount of data to train complex computational models. Therefore, no single method is entirely accurate for nematode detection and quantification. Instead, the choice of methods depends on various factors, including the type of host, sample characteristics, sampling techniques, and the availability of equipment and skilled personnel.

## 4. Conclusions

This review investigated various methods for identifying and quantifying major PPNs and analysed their advantages and limitations. Morphological identification emerged as the most basic approach for taxonomic identification or morphometric characterisation. However, its efficacy is hindered by insufficient morphological traits and microscopic anatomical features, necessitating expertise from nematologists for accurate identification.

Molecular methods can be employed to identify specimens at the genus or species level. Next-generation sequencing is necessary for analysing large volumes of samples. Despite advancements in molecular technologies, morphological methods remain crucial for validating taxonomic identification and providing detailed information on biodiversity. The integration of genetic information from advanced molecular techniques and physical traits information from morphological methods could offer a better approach to identifying PPNs and exploring their interactions with plants and microorganisms.

The emerging technologies are promising, but it is important to evaluate their advantages and limitations before implementing them in agricultural industries. Further, the implementation of machine learning approaches in analysing spectral imaging data and genetic data shows potential in enhancing the identification accuracy and automation of the discovery of genetic markers responsible for parasitism, understanding gene function, and complex patterns in genomic data. In recent years, several studies have generated substantial genomic data, images, and bioinformatic algorithms related to nematodes that need to be managed properly to ensure their accessibility for future studies. Moreover, analysing the genomes of free-living and parasitic nematodes is essential for comprehending nematode parasitism. Further, investigating genetic dysfunctions or abnormalities resulting from the loss of specific genes could be important in understanding nematode parasitism.

## Figures and Tables

**Table 1:** Advantages and disadvantages of molecular markers.

Markers	Advantages	Disadvantages
Restriction fragment length polymorphisms (RFLPs)	Relatively uniform technology, does not required sophisticated tools and prior information about the species [46]. It is simple, fast, and more reliable than isozyme analysis [47].	Irreproducible because of hybridization and digestion [48]. Polymorphism that occurs between rDNA repeats within one species can overlap with the RFLP pattern for another species [49].
Amplified fragment length polymorphism (AFLP)	Highly reproducible, robustness, easy and cost effective, genetic diversity analysis [48]. DNA fragments do not depend on hybridization, partial digestion and faint patterns [50].	Laborious and expensive compared to agarose gel [50]. Requires restriction enzymes, ligation, and adapters.
Random amplification of polymorphic DNA (RAPD)	Simple, rapid, and safe. Useful in detection of intraspecific variation. Reproducible results are obtained using this method [51].	Reproducibility between experiments is relatively low [52]. The method is susceptible to stringent PCR conditions and requires pure DNA from target species [53].
Sequence-characterized amplified region (SCAR)	Does not require restriction enzyme digestion and avoids false positive fragments [54]. Easy, fast and discourages use of radioactive isotopes; beneficial in diagnosing mixed infections. It eliminates the nematode extraction process and the need for prior knowledge of morphology [55].	Random region of gene amplification may lead to uncertainty [56]. Requires advanced laboratory facilities [57].
Random amplified microsatellite polymorphism (RAMP)	High polymorphism. Easy and low cost application [58].	Species specific marker isolation. Unclear mutation mechanism [59].
Inter simple sequence repeat (ISSR)	Highly efficient in studying genetic diversity, genetic fidelity and phylogenetic [60]. Higher reproducibility than RAPD primers [61].	Lower specificity may lead to unclear fingerprint. Low-quality genomic DNA has poor reproducibility [62].
Sequence-related amplified polymorphism (SRAP)	Requires small quantity of template genomic DNA. DNA markers identified without prior genome knowledge [63]. Offers greater potential than the multilocus marker and scalable with next-generation sequencing [64].	Might show less genetic variation [63].

**Table 2:** Identification and enumeration of plant-parasitic nematodes from different hosts using polymerase chain reaction (PCR) and quantitative PCR (qPCR) methods.

Nematode Species	Host	References
*Meloidogyne arabicida*, *Meloidogyne izalcoensis*	Coffee (*Coffea*)	[25]
*Meloidogyne ethiopica*	Kiwi (*Actinidia deliciosa*), Tomato (*Solanum lycopersicum*), Grapevine (*Vitis vinifera*)	[45]
*Meloidogyne chitwoodi*, *Meloidogyne fallax*, *Meloidogyne hapla*	Tomato (*Solanum lycopersicum*)	[52]
*Meloidogyne incognita*, *Pratyenlenchus penetrans*, *Globodera rostochiensis*, *Heterodera glycines*	Radish (*Raphanus sativus*), Sweet potato (*Ipomoea batatas*), Lotus (*Nelumbo nucifera*)	[83]
*Pratylenchus thornei*	Carrot (*Daucus carota*), Wheat	[84]
*Meloidogyne incognita*, *Meloidogyne javanica*, *Meloidogyne arenaria*, *Meloidogyne enterolobii*	Carrot (*Daucus carota*)	[85]
*Pratylenchus vulnus*, *Mesocriconema xenoplax*	Walnut (*Juglans regia*), Almond (*Prunus dulcis*) orchard	[86]
*Rotylenchus reniformis*, *Rotylenchus parvus*, *Heterodera glycines*, *Meloidogyne incognita*	Cotton (*Gossypium herbaceum*), Soybean (*Glycine max*), Banana (*Musa acuminata*), Tobacco (*Nicotiana tabacum*), Eggplant (*Solanum melongena*), Cowpea (*Vigna unguiculata*), Bentgrass (*Agrostis stolonifera*)	[87]
*Pratylenchus alleni* and *Pratyenlenchus penetrans*	Sweet corn (*Saccharata* var. *rugos*)	[88]
*Meloidogyne hapla*	Carrot (*Daucus carota*)	[89]

**Table 3:** Studies on nematode detection using the traditional image analysis method.

Nematode Species	Microscope and Camera	Analysis Method	Test Parameters	References
*Meloidogyne javanica*	Inverted microscope and digital camera	ImageJ, Genstat	R^2^	[111]
*Heterodera glycines*	Kodak Image Station 4000MM Pro (Kodak, NY, USA)	Fluorescence-based imaging system	R^2^	[112]
*Heterodera avenae*	HP Scanjet (HP Inc., CA, USA)	Software KS-400 V.3.0	Accuracy, correlation, variance	[113]
*Meloidogyne incognita*	BX53 Olympus Microscopes (Olympus, Tokyo, Japan)	Python	R^2^, RMSE	[97]

**Table 4:** Detection of plant parasitic nematodes using deep learning.

Nematode Species	Microscope, Camera, Resolution and Input Size	Input Size and Number of Nematodes (NoN)	Deep Learning Models	Test Parameters	References
Genera of *Meloidogyne*, *Pratylenchus Trichodorus*, *Criconema*, *Hemicycliophora*, *Criconemoids*, *Helicotylenchus*, *Hirsmaniella*, *Hoplolaimus*, *Radopholus*, *Trichodorus*, *Xiphinema*	Olympus CX 31 (Olympus, Tokyo, Japan), magnification of 40–100×, Image size (2048 × 1024)	224 × 224, NoN: 1	EfficientNetV2B0, EfficientNetV2M, CoAtNet-0, ResNet101V2	Accuracy, mean class accuracy, F1-score, average precision, average recall	[130]
*Globodera pallida*, *Globodera rostochiensis*, *Globodera mexicana*	Wild Leitz DAPLAN microscope Sony XCD-U100CR (Sony, Tokyo, Japan) with 40× objective lens, Image size (1600 × 1200)	192 × 256, NoN: 1	EB-Net Model	Accuracy, kappa index	[131]
*Ditylenchus*, *Pratylenchus*	Olympus BX51 DIC Microscope (Olympus, Tokyo, Japan), Olympus C5060Wz camera (Olympus, Tokyo, Japan), (10×,100× magnification, Image size (2592 × 1944)	224 × 224, NoN:1	ResNet101	Success rate, misidentified genera	[132]
*Helicotylenchus dihystera*, *Heterodera glycines*, *Pratylenchus brachyourus*	Binocular microscope, Panasonic camera (Panasonic, Osaka, Japan), 4×, 10×, 40×, 100× objective lens, Image size (5120 × 3840)	224 × 224,229 × 229, NoN:1	Xceoption, VGG16, InceptionV3, ResNet, DenseNet, EfficientNet etc	Accuracy, F1-score, precision, recall, specificity	[133]
Root-knot nematode galls (*Meloidogyne* spp.)	Canon EOS 60D camera (Canon Inc, Tokyo, Japan)), Image size (2592 × 1728)	640 × 640, NoN: NA	YOLOv3, YOLOv4, Faster R-CNN, YOLOv5 models	Precision, recall, F1-score, mAP	[134]
*Meloidogyne incognita*	Olympus BX53 Microscope, DP73 Camera (Olympus, Tokyo, Japan), 4×, Objective lens 1200 × 1600	416 × 416, 512 × 512, 614 × 614, NoN: 2~11	YOLOv2-v7	Precision, recall, F1-score, mAP, R^2^, RMSE, CV	[135]
*Meloidogyne incognita*	Olympus BX53 Microscope (Olympus, Tokyo, Japan), DP73 Camera (Olympus, Tokyo, Japan), 4×, Objective lens 1200 × 1600	224 × 224, 480 × 480, 640 × 640, NoN: 2~11	YOLOv5-v7	Precision, recall, F1-score, mAP, R^2^, MAPE	[136]

**Table 5:** Plant-parasitic nematode detection methods based on remote sensing.

Nematode Species	Host	References
*Meloidogyne exigua*, *Meloidogyne paranaesis*, *Meloidogyne incognita*	Coffee (*Coffea arabica*)	[149]
*Heterodera schachtii*, *Rhizoctonia solani*	Sugar beet (*Beta vulgaris*)	[150]
*Bursaphelenchus xylophilus*	Pine tree (*genus Pinus*)	[151]
*Heterodera glycine*, *Meloidogyne incognita*, *Meloidogyne javanica*, *Pratylenchus brachyurus*	Soybean (*Glycine max*)	[152]
*Bursaphelenchus xylophilus*	Pine (*genus Pinus*) trees	[153]
*Meloidogyne incognita*	Cotton (*Gossypium*)	[154]
*Bursaphelenchus xylophilus*	Pine trees (*genus Pinus*)	[155]
*Heterodera schachtii*	Sugar beet (*Beta vulgaris*)	[156]
*Globodera pallida*, *Globodera rostochiensis*	Potato (*Solanum tuberosum*)	[157]
*Meloidogyne*	Lettuce (*Lactuca sativa*)	[158]
*Bursaphelenchus Xylophilus*	Pine trees (*genus Pinus*)	[159]
*Pratylenchus thornei*	Wheat (*Triticum*)	[160,161]

## Data Availability

All the data are included the in manuscript.

## Abbreviations

Abbreviations	Meaning
AFLP	Amplified fragment length polymorphism
ANN	Artificial neural network
BKW	Basal knobs width
BKTH	Basal knobs to head length
CBAM-CA	Convolution block attention module-coordinate attention
CC	Chlorophyll content
CCVA	Custom computer vision algorithm
CIoU	Complete intersection over union
CNN	Convolution neural network
COI	Cytochrome oxidase subunit I
Ct	Cycle threshold
DEGO	Dorsal oesophageal gland orifice
DFNN	Deep feedforward neural network
DNA	Deoxyribonucleic acid
FTA	Flinders Technology Associates
GPS	Geographical positioning system
IGS	Intergenic spacer region
ITS	Internal transcribed spacers
IDW	Inverse distance weighting
J2	Second-stage juvenile
LAI	Leaf area index
LDA	Linear discriminant analysis
MALDI-TOFMS	Matrix-assisted laser desorption/ionisation time-of-flight mass spectrometry
mtDNA	Mitochondrial DNA
mtMG	Mitochondrial metagenomics
NDVI	Normalised difference vegetation index
NGS	Next-generation sequencing
NIR	Near-infrared
PCA	Principal component analysis
PCR	Polymerase chain reaction
PLS-DA	Partial least square discriminant analysis
PLS-SVM	PLS support vector machine
PPNs	Plant-parasitic nematodes
PWD	Pine wilt diseases
ddPCR	Droplet digital PCR
qPCR	Quantitative polymerase chain reaction
rDNA	Recombinant DNA
RAPD	Random amplification of polymorphic DNA
RFLP	Restriction fragment length polymorphism
RKN	Root-knot nematode
RNN	Recurrent neural network
rRNA	Ribosomal RNA
SAM	Spectral angle mapper
satDNA	Satellite DNA
SCAR	Sequence-characterized amplified region
SCN	Soybean cyst nematodes
SIPI	Structural independent pigment index
SPAD	Spoil and plant development analyser
SRPI	Simple ratio pigment index
SVM	Support vector machine
t-SNE	t-distributed stochastic neighbour embedding
YOLO	You only look once
